# Linear ubiquitination of p31^comet^ by HOIP couples cytokine response with mitotic regulation

**DOI:** 10.1186/s13578-025-01416-8

**Published:** 2025-06-03

**Authors:** Yifeng Gao, Qing Yin, Yaser Gamallat, Michael G. Grant, Aidan H. Snell, Xingxing Shi, Lara N. Ulstad, Arshita Singh, Tingan Chen, Joseph O. Johnson, Dorina Avram, Lixin Wan

**Affiliations:** 1 Department of Molecular Oncology, H. Lee Moffitt Cancer Center and Research Institute, Tampa, FL 33612 USA; 2 Department of Molecular Biosciences, College of Arts and Sciences, University of South Florida, Tampa, FL 33620 USA; 3 Department of Chemistry, College of Arts and Sciences, University of South Florida, Tampa, FL 33620 USA; 4 Analytic Microscopy Core Facility, H. Lee Moffitt Cancer Center and Research Institute, Tampa, FL 33612 USA; 5 Department of Immunology, H. Lee Moffitt Cancer Center and Research Institute, Tampa, FL 33612 USA; 6 Department of Cutaneous Oncology, H. Lee Moffitt Cancer Center and Research Institute, Tampa, FL 33612 USA

**Keywords:** Mitotic checkpoint complex, P31^comet^, Spindle assembly checkpoint, Anaphase-promoting complex, CDC20, HOIP, LUBAC, Proinflammatory cytokine

## Abstract

**Background:**

Inflammation and genomic instability are among the hallmarks of human cancer. Proinflammatory cytokines induce DNA damage through the accumulation of reactive oxygen and nitrogen species (RONS), which often leads to base alternations. The link between proinflammatory cytokines and chromosomal instability remains largely elusive.

**Results:**

Here, we report that the mitotic checkpoint protein p31^comet^ (MAD2L1BP) is modified by linear ubiquitination via the E3 ubiquitin ligase HOIP after cytokine stimulation. HOIP-mediated polyubiquitination of p31^comet^ occurs on its C-terminal lysine residues. Ubiquitinated p31^comet^ displays reduced binding to PLK1, which phosphorylates and inactivates p31^comet^. Thus HOIP positively  regulates p31^comet^ function. Consistent with this notion, HOIP-deficient cells exhibit prolonged mitotic duration similar to p31^comet^ knockout. Mitotic defects are also more prevalent in cells without HOIP or p31^comet^. Moreover, compared with the cells expressing wild-type p31^comet^, cells expressing a ubiquitination-deficient p31^comet^ mutant take more time to complete the M phase.

**Conclusions:**

Our results together uncover a mechanistic link between the proinflammatory cytokines and the mitotic checkpoint pathways. This molecular switch could be explored as a potential therapeutic target in inflammation-driving or p31^comet^ overexpressed cancer types.

**Supplementary Information:**

The online version contains supplementary material available at 10.1186/s13578-025-01416-8.

## Background

The spindle assembly checkpoint (SAC) ensures accurate chromosome segregation during mitosis, which is essential for maintaining genomic stability [1]. The SAC is established in prometaphase and remains active until all the kinetochores are properly attached to the microtubules [2]. One of the key roles of an active SAC in eukaryotic cells is to inhibit the anaphase promoting complex/cyclosome (APC/C or APC), a ubiquitin E3 ligase that drives metaphase to anaphase transition through degrading mitotic cyclins and the separase inhibitor securin [3]. SAC-mediated inhibition of APC/C is achieved through the engagement of the mitotic checkpoint complex (MCC) consisting of MAD2 (human gene symbol MAD2L1), BUBR1(human gene symbol BUB1B), BUB3, and the APC/C co-activator CDC20 [4]. MCC directly binds to APC/C to suppress its enzymatic function [5]. The assembly of a functional MCC relies on the interaction between MAD2 and CDC20 [6], which is in turn regulated by the kinetochore-bound SAC protein MAD1 [7]. MAD1-bound MAD2 adopts a closed conformation (C-MAD), which facilitates the transformation of an open, inactive, MAD2 conformer (O-MAD2) to an intermediate state (I-MAD2) [8, 9]. The I-MAD2 then interacts with CDC20 followed by further conversion to a closed conformer (C-MAD2). The C-MAD2-CDC20 complex recruits BUBR1 and BUB3 to form the MCC complex [10].

Upon SAC satisfaction in metaphase, the functional MCC complex needs to be disassembled to unleash APC/C for the anaphase onset. p31^comet^ (human gene symbol MAD2L1BP), a MAD2 structural homolog, competes with O-MAD2 to bind C-MAD2 thereby hindering efficient MCC assembly [11, 12]. Hence, it is not surprising that p31^comet^-depleted cells arrest in metaphase despite a satisfied SAC [13, 14]. p31^comet^ expression in mammalian cells has been suggested to be controlled by E2 F family transcription factors, therefore is often found higher in tumor samples compared with normal tissues [15]. Different from other cell cycle proteins, p31^comet^ is relatively stable across the cell cycle [15], suggesting that p31^comet^ function is primarily modified through non-proteolytic post-translational mechanisms. Indeed, PLK1 phosphorylates p31^comet^ on S102 to disrupt p31^comet^-MAD2 interaction to maintain an active SAC [16]. In contrast to PLK1, in Xenopus eggs, IKKβ phosphorylates p31 on S4, T6, and T179 to facilitate its binding to MAD2 [17]. Whether p31^comet^ is subjected to other types of post-translational modifications remains undetermined.

We present here that p31^comet^ is poly-ubiquitinated by the ubiquitin E3 ligase HOIP in cells. HOIP (human gene symbol RNF31), the main catalytic subunit of the linear ubiquitin chain assembly complex (LUBAC), is the only E3 ligase that assembles the linear (or M1-linked) poly-ubiquitin chains [18]. LUBAC plays an essential role in mediating the canonical NF-κB pathway activation after proinflammatory cytokine stimulation. LUBAC catalyzes the linear ubiquitination of NEMO, RIP1, and FADD [19]. The linear poly-ubiquitin chains formed on these molecules function as a scaffold to recruit IκB kinases (IKKs) and subsequently lead to the nuclear localization and activation of NF-κB transcription factors [19]. We found that similar to p31^comet^ knockdown, depletion of HOIP in cells delayed mitotic progression, indicating a positive regulation of p31^comet^ function by HOIP. Consistently, accumulated APC/C substrates were found in HOIP and p31^comet^-deficient cells. Moreover, cells expressing a ubiquitination-deficient p31^comet^ mutant exhibited a slower mitotic progression. These findings hence provide a mechanism for the observed cytokine-stimulated mitotic progression in tumor cells.

## Methods

### Plasmids

List of the plasmids used in this study can be found in the **Key Resources Table** in the section.

p31^comet^, MAD1, MAD2, BUBR1, NEMO, and TRIP13 cDNAs were subcloned into pFlag-CMV2, pcDNA3-HA, pGEX-4 T-1, pET28a, and pLenti-CMV-hygro vectors. pLKO.1-puro and lentiCRISPRv2-puro constructs were used to generate shRNA and sgRNA lentiviral vectors. The targeting sequences of sh*HOIP-*A, B, C are 5’-GGCGTGGTGTCAAGTTTAA-3’, 5’-CCTAGAACCTGATCTTGCA-3’, 5’-GCTGTGCTATGTTCCATAT-3’. The targeting sequences of sh*HOIL-1L-*A, B are 5’-TTTGACAGATGAGTGTTGTGG-3’, 5’-AAGGAATCCCATTTACCCTGC-3’. The targeting sequences of sh*SHARPIN-*A, B, C *are* 5’-TCGGATGGTGTAGGAAACTGA-3’, 5’-ATGAAGGTGCAGGAAGGACAG-3’, 5’-TCTCCGTCAAGTTTCCAGGGC-3’. The targeting sequences of sh*p31-*A, B *are* 5’-TGTGCCATGACGACGGTGCCC-3’, 5’-AAGCGTTGAGAGGTTCCTGCG-3’. The targeting sequence of sg*HOIP* is 5’-GCCCTCAGCGGCCTCGGTAC-3’, and the targeting sequence of sg*p31* is 5’-CTCAAGTAGTTCTATCTGGG-3’.

### Antibodies

List of the antibodies used in this study can also be found in the **Key Resources Table** in the section.

Anti-securin (13,445, 1:1000), anti-HOIP/RNF31 (99,633, 1:1000), anti-Ubiquitin (43,124, 1:1000), anti-Aurora A (14,475, 1:1000), anti-MAD2 (4636, 1:1000), anti-p-NF-κB p65 (3033, 1:1000), anti-NF-κB p65 (8242, 1:2000), anti-SHARPIN (12,541, 1:2000), anti-p-IκBa (2859, 1:2000), anti-IκBa (4814, 1:1000), anti-Bim (3339, 1:2000), anti-p-S139-H2 A.X (9718, 1:2000), and anti-GST (2622, 1:2000) antibodies were purchased from Cell Signaling Technology. Anti-cyclin B1 (GNS1, 1:2000), anti-Cdc20 (p55 CDC E-7, 1:2000), anti-c-Myc (9E10, 1:2000) and polyclonal anti-HA (Y-11, 1:2000) antibodies were purchased from Santa Cruz. Anti-p31^comet^ (clone E29.19.14, MABE451), anti-HOIL-1L (clone 2E2 MABC576, 1:2000), anti-TUBULIN (T-5168, 1:2000), anti-VINCULIN (V-4505, 1:2000), polyclonal anti-Flag antibody (F-2425, 1:2000), monoclonal anti-Flag (F-3165, 1:2000) antibody, anti-Flag agarose beads (A-2220), and anti-HA agarose beads (A-2095), peroxidase-conjugated anti-mouse secondary antibody (A-4416, 1:3000) and peroxidase-conjugated anti-rabbit secondary antibody (A-4914, 1:3000) were purchased from Sigma. Monoclonal anti-HA antibody (MMS-101P, 1:2000) was purchased from Covance.

### Cell culture, transfection and synchronization

293T, HEK293, HeLa, HCT116, DLD1, and U2-OS cell lines were obtained from ATCC and grown in Dulbecco’s Modified Eagle Medium (DMEM) supplemented with 10% fetal bovine serum (FBS), 2 mM Glutamine, 100 units∙ml^−1^ penicillin and 100 mg∙ml^−1^ streptomycin. Transfection of plasmids into 293T follows the PEI transfection as described in https://www.addgene.org/protocols/transfection. All cell lines were routinely tested to be negative for mycoplasma contamination. Cell synchronization using Nocodazole arrest and release has been described previously [20].

### Site-directed mutagenesis

Site-directed mutagenesis to generate HOIP and p31^comet^ mutants was performed using the QuikChange XL Site-Directed Mutagenesis Kit (Agilent) according to the manufacturer’s instructions.

### Lentiviral and retroviral packaging and transduction

Lentiviral constructs were co-transfected with the pCMV-dR8.91 (Delta 8.9) plasmid containing *gag*, *pol* and *rev* genes and the VSV-G envelope-expressing plasmid into 293 T cells. For packaging retrovirus, retroviral constructs were co-transfected with VSV-G, JK3, and pCMV-tat into 293 T cells. Virus-containing media were collected, and filtered before being used for transduction [21]. The transduced cells were selected with 1 μg∙ml^−1^ puromycin for at least 48 to72 h before harvest or downstream analyses.

### Immunoblots and immunoprecipitation

Cells were lysed in EBC buffer (50 mM Tris pH 7.5, 120 mM NaCl, 0.5% NP-40) supplemented with protease inhibitors (Thermo Scientific) and phosphatase inhibitors (Thermo Scientific). To prepare the Whole Cell Lysates (WCL), 3 × SDS sample buffer was directly added to the cell lysates and sonicated before being resolved on SDS-PAGE and subsequently immunoblotted with primary antibodies. The protein concentrations of the lysates were measured using the Bio-Rad protein assay reagent on a Bio-Rad Model 680 Microplate Reader. For immunoprecipitation, 1 mg lysates were incubated with the appropriate agarose-conjugated primary antibody for 3–4 h at 4 °C or with unconjugated antibody (1–2 μg) overnight at 4 °C followed by 1 h incubation with Protein G Sepharose beads (GE Healthcare). Immuno-complexes were washed four times with NETN buffer (20 mM Tris, pH 8.0, 100 mM NaCl, 1 mM EDTA and 0.5% NP-40) before being resolved by SDS-PAGE and immunoblotted with indicated antibodies.

### In vitro binding assays

The pGEX-4 T-1-p31 plasmid was transformed into BL21(DE3) competent cells. The recombinant GST-p31 ITCH proteins were expressed by Isopropyl β-D-1-thiogalactopyranoside (IPTG) induction for 18 h at 16 °C. The proteins were purified using Glutathione Sepharose 4B (GE Healthcare) according to the manufacturer’s instructions. Agarose-bound GST and GST-p31 proteins were further incubated with Flag-HOIP-expressing 293 T lysates. GST-pull down experiments were also performed by incubating GST-fusion proteins with cell lysates from 293 T cells expressing the indicated exogenous proteins. The incubation was performed at 4 °C for 3–4 h followed by washing with NETN buffer as described in the ***Immunoblots and Immunoprecipitation*** section above. Samples were resolved by SDS-PAGE and subjected to immunoblot analysis.

### Cell-based in vitro ubiquitination assay

293 T cells were transfected with Flag-HOIP, His-ubiquitin and HA-p31 plasmids. 48 h after transfection, cells were harvested in denatured buffer (6 M guanidine-HCl, 0.1 M Na_2_HPO_4_/NaH_2_PO_4_, 10 mM imidazole), followed by Ni–NTA (Ni-nitrilotriacetic acid) purification and immunoblot analysis.

### In vitro ubiquitination assay

His-p31 and His-NEMO proteins were expressed and purified in Rosetta (DE3) *E. coli*. Flag-HOIP proteins were immunopurified from 293 T cells transfected with the Flag-HOIP plasmid, the proteins were immobilized to the Flag M2 agarose beads and washed three times with NETN buffer containing 500 mM NaCl. In vitro ubiquitination assay was performed in 30 μl ubiquitination assay buffer (20 mM HEPES pH 7.2, 5 mM MgCl_2_, 0.1 mM DTT, 1 mM ATP), with 50 nM E1, 500 nM UBCH7 (E2), 1 μg of ubiquitin or ubiquitin variants (Boston Biochem). 500 ng GST-ITCH (wild-type or mutant) together with 500 ng His-BRAF or immuno-purified Flag-BRAF proteins were added to the buffer to initiate the reaction. Samples were incubated at 30 °C for 60 min. The reactions were stopped by the addition of 2 × SDS-PAGE sample buffer, and the reaction products were resolved by SDS-PAGE and probed with indicated antibodies [22, 23].

### Time-lapse microscopy

Time lapse microscopy experiments of YFP-H2B/HeLa cells grown in 6 well plates were performed with either an Incucyte S3 or SX5 (Sartorius AG, Gottingen, Germany) live cell imaging or Opera Phenix Plus High Content Screening (Revvity, Inc., Massachusetts, USA) system. On the Incucyte systems 9 fields from each well were captured every 5 min for 24 h with a 20 × objective lens. Images were created with LED based phase contrast and green fluorescence (S3: 440–480 nm excitation and 504–544 nm emission, SX5:453–485 nm excitation and 494–533 nm emission) channels. Images were exported to uncompressed TIFF file format and analyzed with ImageJ. Similarly, the Opera Phenix was used to capture 9 adjacent fields from each well, with 5% overlap for improved stitching, at 5-min intervals for 24 h. The images were captured in non-confocal mode with a 20X/0.4 NA lens, correction collar set to 1.0 mm, using brightfield and green fluorescence (488 nm laser excitation, 500–550 nm emission filter) channels. Raw image Tiff files were exported and analyzed with ImageJ. Mitosis duration was calculated as the time from the first image showing nuclear envelope breakdown (NEBD) to the first image showing nuclear envelope reformation.

### Flow cytometry

Cells were trypsinized and re-suspended in 200 μl cold PBS, 5 ml of cold 90% ethanol was added for fixation overnight. Prior to the assay, cells were centrifuged for 5 min at 200 × g and re-suspended in 0.5 ml PBS with propidium iodide (PI, 50 μg/ml, EMD Millipore) and RNase A (250 μg/ml, Roche). After incubating 30 min at 37 °C, cells were transferred into FACS tubes and analyzed using a BD FACSCanto II flow cytometer. The results were analyzed using the FlowJo software.

### Immunofluorescence

Cells were seeded onto coverslips in 6-well plates. Prior to the experiments, cells were fixed with ice-cold methanol for 20 min, followed by incubation with 0.1% Triton X-100 in PBS for 10 min. Cells were pre-blocked with 2% BSA/PBS for 45 min, then incubated with primary antibodies overnight at 4 °C and followed by secondary antibodies conjugated with Alexa-Fluor-488 or Alexa-Fluor-594. DAPI was used to stain the nuclei. Microscopic images were obtained using a Leica SP8 Confocal Microscope.

### Statistical analyses

All quantitative data were presented as the mean ± SEM (standard error of the mean) or the mean ± SD (standard deviation) as indicated of at least three independent experiments by Student’s t-test for between-group differences. The *P* < 0.05 was considered statistically significant.

### Key resources table

**Table:** 

Reagent	Source	Identifier
*Antibodies*		
securin	Cell Signaling Technology	13,445
HOIP/RNF31	Cell Signaling Technology	99,633
Ubiquitin	Cell Signaling Technology	43,124
Aurora A	Cell Signaling Technology	14,475
MAD2	Cell Signaling Technology	4636
p-NF-κB p65	Cell Signaling Technology	3033
NF-κB p65	Cell Signaling Technology	8242
SHARPIN	Cell Signaling Technology	12,541
p-IκBa	Cell Signaling Technology	2859
IκBa	Cell Signaling Technology	4814
Bim	Cell Signaling Technology	3339
GST	Cell Signaling Technology	2622
p-S139-H2 A.X	Cell Signaling Technology	9718
cyclin B1	Santa Cruz Biotechnology	GNS1
Cdc20	Santa Cruz Biotechnology	p55 CDC E-7
c-Myc	Santa Cruz Biotechnology	9E10
HA (polyclonal)	Santa Cruz Biotechnology	Y-11
HA (monoclonal)	Covance	MMS-101P
p31^comet^	MilliporeSigma	clone E29.19.14, MABE451
HOIL-1L	MilliporeSigma	clone 2E2 MABC576
TUBULIN	MilliporeSigma	T-5168
VINCULIN	MilliporeSigma	V-4505
Flag (polyclonal)	MilliporeSigma	F-2425
Flag (monoclonal)	MilliporeSigma	F-3165
anti-Flag agarose beads	MilliporeSigma	A-2220
anti-HA agarose beads	MilliporeSigma	A-2095
peroxidase-conjugated anti-mouse secondary antibody	MilliporeSigma	A-4416
peroxidase-conjugated anti-rabbit secondary antibody	MilliporeSigma	A-4914
*Plasmids*		
pCMV3flag8HOIP	Addgene	50,015
pCMV3flag8HOIL-1L	Addgene	50,016
pCMV3-flag8-SHARPIN	Addgene	50,014
pcDNA5 FRT TO myc p31 Comet	Addgene	59,833
pERB160-hMAD1	Addgene	58,285
pcDNA5-EGFP-AID-BubR1	Addgene	47,330
VL3 (Flag-Mad2)	Addgene	16,047
pcDNA3 HA human NEMO	Addgene	13,512
pLKO.1-puro	Addgene	8453
lentiCRISPRv2-puro	Addgene	98,290
pHK-mTRIP13	Dr. Kevin D. Corbett, UCSD	
pcDNA3-6His-ubiquitin and mutants	PMID: 31,015,455	

## Results

### HOIP positively regulates APC/C^Cdc20^

**Fig. 1:**
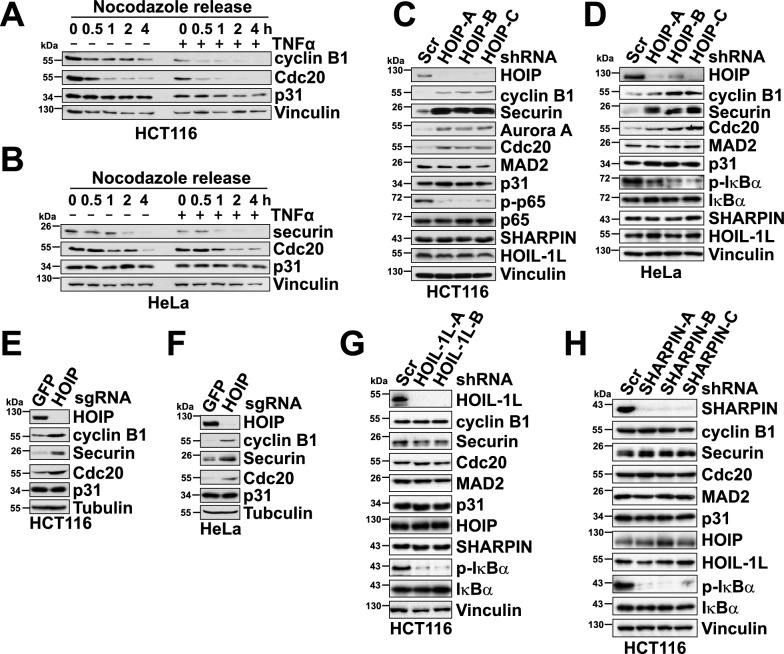
APC/C^Cdc20^ substrates are accumulated in HOIP-depleted cells. **A–B** Immunoblot (IB) analysis of whole-cell lysates (WCL) derived from HCT116 (**A**) and HeLa (**B**) cells. Cells were arrested in mitosis using 300 nM nocodazole for 16 h. Mitotic cells were collected by mechanical shake-off and released back into the cell cycle in media without nocodazole. Cells were harvested at the indicated time points after nocodazole release. When indicated, cells were treated with 50 ng∙ml^−1^ TNFα when released from nocodazole release. **C-D.** IB analysis of WCL derived from HCT116 (**C**) and HeLa (**D**) cells transduced with shScr (as the negative control) or the indicated lentiviral sh*HOIP* constructs. **E–F** IB analysis of WCL derived from HCT116 (**E**) and HeLa (**F**) cells transduced with sgGFP (as the negative control) or sg*HOIP* lentiviral constructs. **G–H** IB analysis of WCL derived from HCT116 cells transduced with shScr (as the negative control) or the indicated lentiviral sh*HOIL-1L* (**G**) or sh*SHARPIN* (**H**) constructs. (See also Figure S1)

Proinflammatory cytokines, such as TNFα, IL-1β, and IL-6, produced after pathogen infection or tissue damage, contribute to mounting an effective immune response [24]. On the other hand, these cytokines exhibit tumor-promoting functions through a wide variety of mechanisms [25]. Our previous study uncovered a growth-promoting role of proinflammatory cytokines in melanoma cells through JNK-ITCH-mediated non-proteolytic ubiquitination of the BRAF kinase [26]. Proinflammatory cytokines have also been well-documented for their roles in provoking genomic instability [27]. One example is the increased reactive oxygen species (ROS) during inflammatory responses. ROS causes DNA damage, including DNA crosslinks and strand breaks [28]. Aside from a direct impact on DNA, proinflammatory cytokines have also been suggested to promote chromosome instability [29]. Since a compromised spindle assembly checkpoint is the leading cause of chromosome instability [30], we examined the roles of TNFα in mitotic exit using nocodazole arrest and release. Compared with control cells, TNFα treatment of HCT116, HeLa, and HEK293 cells accelerated mitotic exit as evidenced by enhanced degradation of cyclin B1 and securin after the release (Figs. 1A–B, S1 A-F). These results indicate that proinflammatory cytokines including TNFα and IL-1β potentially facilitate the activation of APC/C^Cdc20^ by compromising the spindle assembly checkpoint.

Cytokines, including TNFα, drive NF-κB pathway activation by engaging the ubiquitin pathway to facilitate signal transduction, a process in which the LUBAC complex plays an indispensable role [18]. A recent report highlights the role of LUBAC in chromosome congression and alignment through linear ubiquitination of CENP-E [31]. To assess whether the LUBAC complex modulates mitotic exit, HCT116, HeLa, HEK293, DLD1, and U2-OS cells were transduced with lentiviral shRNA vectors targeting HOIP. We found that compared with the control cells, HOIP knockdown using 3 different shRNAs led to the accumulation of the APC/C^Cdc20^ substrates cyclin B1, securin, Aurora A, Bim, and Cdc20 (Figs. 1C–D, S1G-I). Likewise, cyclin B1 and securin were upregulated in HOIP knockout cells generated by CRISPR/Cas9 (Figs. 1E–F, S1 J). In contrast, these APC/C^Cdc20^ substrates remained unchanged after depletion of HOIL-1L or SHARPIN, the other two LUBAC subunits (Figs. 1G–H, S1 K-N), although the knockdown suppressed p-S32-IκBα and p-S536-p65/RelA, markers associated with NF-κB activation (Figs. 1G–H, S1 K-N). This observation also indicates that accumulated APC/C^Cdc20^ substrates are not caused by NF-κB activation following HOIP depletion (Figs. 1C–D, S1G-I). We further examined the levels of the mitotic checkpoint proteins, including MAD2 and p31^comet^, and found that they were not affected by HOIP, HOIL-1L, or SHARPIN knockdown (Figs. 1C-D, E–F, S1G-I, K-N). These results support the notion that HOIP, but not other LUBAC components, exert a positive regulation of APC/C^Cdc20^ activity.

### HOIP, but not the other LUBAC subunits, interacts with p31^comet^

**Fig. 2:**
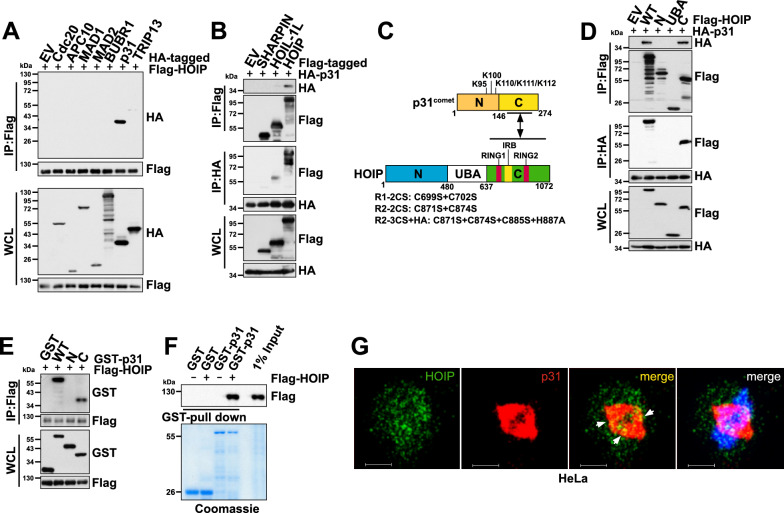
HOIP specifically binds to p31^comet^. **A**. Immunoblot (IB) analysis of whole-cell lysates (WCL) and anti-Flag immunoprecipitates (IP) derived from asynchronous 293 T cells transfected with Flag-HOIP and the indicated HA-tagged constructs. EV: empty vector. **B**. IB analysis of WCL, anti-HA, and anti-Flag IP derived from asynchronous 293 T cells transfected with HA-p31^comet^ and the indicated Flag-tagged constructs. **C**. Domain structures of p31^comet^ and HOIP. The ubiquitinated lysines are indicated in the p31^comet^ domain structure. Functional domains are indicated in the HOIP domain structure. Catalytically inactive HOIP mutants are indicated at the bottom. **D**. IB analysis of WCL, anti-HA, and anti-Flag IP derived from asynchronous 293 T cells transfected with HA-p31^comet^ and the indicated Flag-HOIP constructs. **E**. IB analysis of WCL and anti-Flag IP derived from asynchronous 293 T cells transfected with Flag-HOIP and the indicated HA-p31^comet^ constructs. **F**. p31^comet^ bound to HOIP in vitro. Immunopurified Flag-HOIP was incubated with the indicated recombinant GST proteins before washing and being resolved by SDS-PAGE and IB. **G**. Immunofluorescent staining of HeLa cells using anti-HOIP and anti-p31^comet^ antibodies, DAPI was used for DNA staining. Representative images shown here are from a mitotic HeLa cell. (See also Figure S2)

To search for the protein(s) tethered by HOIP to the MCC-APC/C machinery, we compared the binding of HOIP to a panel of APC/C and MCC components. As shown in Fig. 2A, HOIP specifically interacted with p31^comet^, but not with Cdc20, APC/C subunit APC10, MCC subunits MAD2, BUBR1, or the MAD2 conformation modulating enzyme TRIP13 [32]. On the other hand, p31^comet^ primarily bound to HOIP, and to a much lesser extent, to HOIL-1L, but not to SHARPIN (Fig. 2B), consistent with the observation that silencing HOIL-1L or SHARPIN had no effect on the levels of APC/C^Cdc20^ substrates (Figs. 1E–F, S1E-H). Co-immunoprecipitation experiments revealed that p31^comet^ bound to the C-terminal region of HOIP, which contains the catalytic RING-between-RING (RBR) domain (Fig. 2C–D). Moreover, we found that the C-terminal of p31^comet^ interacted with HOIP (Fig. 2C and E), which is on the opposite side of the MAD2-p31^comet^ binding interface [12]. To rule out the possible indirect binding between HOIP and p31^comet^, recombinant GST-p31^comet^ proteins were purified from *E.coli* for GST pull-down analysis. As shown in Fig. 2F, we observed strong binding of GST-p31^comet^ and Flag-HOIP proteins in vitro, the latter derived from Flag-HOIP-transfected 293 T cells. To further determine if p31^comet^ and HOIP are co-localized in cells, we performed immunofluorescence analysis using HeLa cells. We were particularly interested in comparing their subcellular localizations in mitotic and interphase cells. As demonstrated in Figs. 2G and S2A, HOIP localization was widely distributed in mitotic cells. Intriguingly, p31^comet^ was primarily co-localized with microtubules, as reported previously [33]. Despite the difference in their subcellular localizations, numerous colocalizations were observed near the metaphase plate (Figs. 2G and S2 A). In contrast, in interphase HeLa cells, p31^comet^ was mainly found in the cytosol while HOIP was primarily found in the nucleus, they exhibit a minimal overlap in their localizations (Figure S2B).

### HOIP promotes linear poly-ubiquitination of p31^comet^

**Fig. 3:**
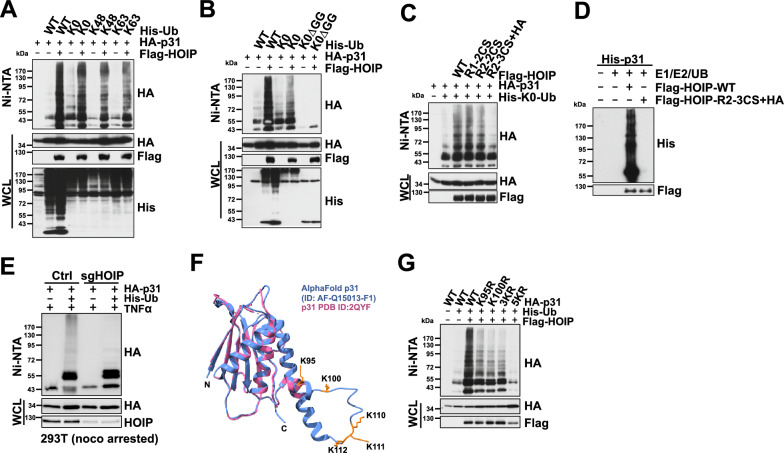
HOIP promotes linear ubiquitination of p31^comet^. **A.** Immunoblot (IB) analysis of whole cell lysates (WCL) and Ni–NTA (Ni-nitrilotriacetic acid) affinity precipitates derived from 293T cells transfected with HA-p31^comet^, Flag-HOIP and the indicated His-UB constructs. The indicated lysine residue denotes that all but the indicated lysine (K) was mutated to arginine (R). K0: all the seven lysines of the ubiquitin were mutated to arginine. **B**. IB analysis of WCL and Ni–NTA affinity precipitates derived from 293T cells transfected with HA-p31^comet^, Flag-HOIP and the indicated His-UB constructs. K0-ΔGG denotes the K0-UB with the two C-terminal glycine (G) residues deleted. **C**. IB analysis of WCL and Ni–NTA affinity precipitates derived from 293T cells transfected with HA-p31^comet^, His-UB, and the indicated Flag-HOIP constructs. **D**. Bacterially purified recombinant His-p31^comet^ proteins were incubated with E1, E2, ubiquitin (UB), immune-purified WT, and catalytic inactive (R2-3 CS + HA) Flag-HOIP as indicated at 30 °C for 60 min before resolved by SDS-PAGE and IB analysis. **E**. IB analysis of WCL and Ni–NTA affinity precipitates derived from control (Ctrl) and sgHOIP-293T cells transfected with HA-p31^comet^ and His-UB constructs as indicated. 293T cells were treated with 300 nM nocodazole for 16 h. Mitotic cells were collected by mechanical shake-off. **F**. Crystal structure of p31^comet^ (PDBID 2QYF) was superimposed with the AlphaFold predicted structure to highlight the localization of the C-terminal lysine residues. **G**. IB analysis of WCL and Ni–NTA affinity precipitates derived from 293 T cells transfected with Flag-HOIP, His-UB, and the indicated HA-p31^comet^ constructs. (See also Figure S3)

HOIP is the major catalytic subunit for the LUBAC complex. We found that HOIP could efficiently promote the poly-ubiquitination of p31^comet^ regardless of the three ubiquitin (UB) mutants used (Fig. 3A), suggesting the poly-ubiquitin chains assembled on p31^comet^ are predominantly M1-linked. Indeed, when the two carboxyl glycine residues were deleted from the UB molecule [34], the mutant UB could not assemble the poly-ubiquitin chain on the p31^comet^ protein (Fig. 3B). HOIP belongs to the RBR family E3 ligases, which contain two catalytically active RING domains [35]. The RING1 domain in HOIP interacts with UB-loaded E2, while a cysteine residue on the RING2 domain accepts the UB to facilitate its transfer to substrate proteins. The cysteine/histidine residues on both RING domains are essential for HOIP autoubiquitination in vitro [36]. To assess if these catalytic cysteines are important for the ubiquitination of p31^comet^, these cysteines and histidines were mutated to serine and alanine, respectively (Fig. 2C). Although mutating the cysteines on the RING1 domain to serines (C699S + C702S, termed R1-CS) failed to block HOIP’s ability to ubiquitinate p31^comet^, the substitution of RING2 cysteine key residues (C871S, C874S, C885S and H887 A, termed R2-3 CS + HA) substantially suppressed HOIP-mediated p31^comet^ poly-ubiquitination (Fig. 3C). Moreover, we found that similar to the well-characterized HOIP substrate NEMO [37] (Figure S3 A), p31^comet^ could be efficiently ubiquitinated in vitro by affinity-purified WT-HOIP but not by the R2-3 CS + HA mutant (Fig. 3D). This observation supports the notion that HOIP directly promotes p31^comet^ ubiquitination. Next, we collected mitotic arrested control and sgHOIP-293T cells transfected with HA-p31^comet^ and His-UB to compare p31^comet^ ubiquitination in mitotic cells after TNFα stimulation. HOIP depletion almost completely eliminated p31^comet^ ubiquitination (Fig. 3E), further depicting the essential role of HOIP as a key enzyme to regulate p31^comet^ ubiquitination in M phase cells.

There are 12 lysines in the p31^comet^; we reasoned that the ubiquitinated lysine(s) could be found in the disordered region of p31^comet^, not resolved by the crystal structure (Fig. 3F). Mutation of K95, K100, or K110, K111, K112 (3KR) significantly compromised HOIP-mediated p31^comet^ ubiquitination. When all five lysines were mutated to arginine (5KR), HOIP failed to promote p31^comet^ ubiquitination (Fig. 3G). These results coherently demonstrate that HOIP catalyzes the linear ubiquitination of p31^comet^ at its C-terminal lysine residues.

### HOIP accelerates mitotic progression

**Fig. 4:**
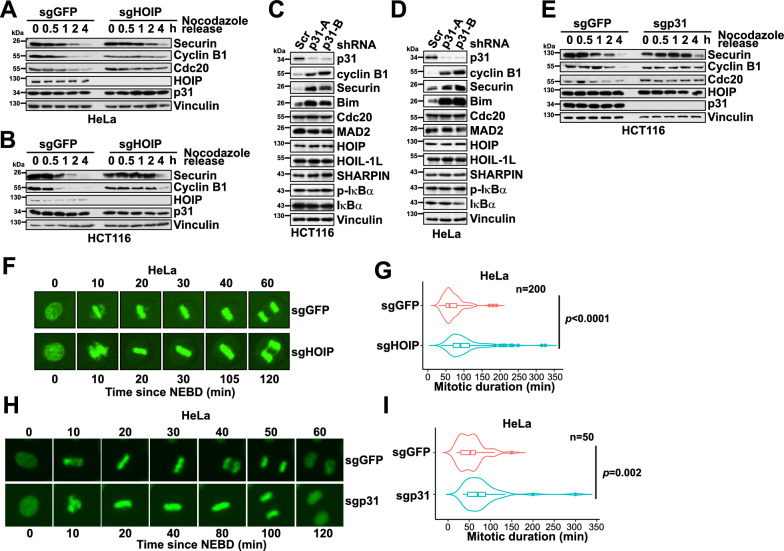
Delayed mitotic exit is observed in HOIP-depleted cells. **A–B** Immunoblot (IB) analysis of whole-cell lysates (WCL) derived from control (sgGFP) or HOIP-deleted (sgHOIP) HeLa (**A**) and HCT116 (**B**) cells. Cells were arrested in mitosis using 300 nM nocodazole for 16 h. Mitotic cells were collected by mechanical shake-off and released back into the cell cycle in media without nocodazole. Cells were harvested at the indicated time points after nocodazole release. **C–D**. IB analysis of WCL derived from HCT116 (**C**) and HeLa (**D**) cells transduced with shScr (as the negative control) or the indicated lentiviral sh*p31* constructs. **E.** IB analysis of WCL derived derived from control (sgGFP) or p31^comet^-deleted (sgp31) HCT116 cells. Cells were arrested in mitosis using 300 nM nocodazole for 16 h. Mitotic cells were collected by mechanical shake-off and released back into the cell cycle in media without nocodazole. Cells were harvested at the indicated time points after nocodazole release. **F–G.** Time-lapse microscopy of sgGFP and sgHOIP HeLa cells stably expressing a H2B-YFP retroviral construct. **F** Representative images showing that sgHOIP-HeLa took a significantly longer time to complete the mitosis. **G** Quantification of the recorded mitotic duration times (minutes) of sgGFP and sgHOIP cells, n = 200, Student’s t-test. **H–I.** Time-lapse microscopy of sgGFP and sgp31 H2B-YFP-HeLa cells. **H** Representative images showing that sgp31-HeLa took a significantly longer time to complete the mitosis. **I** Quantification of the recorded mitotic duration times (minutes) of sgGFP and sgHOIP cells, n = 50, Student’s t-test. (See also Figure S4)

In accordance with the positive role of HOIP in activating APC/C^Cdc20^, *HOIP* knockout HeLa and HCT116 cells exhibited a prolonged mitotic exit after the release from Nocodazole arrest, compared with control cells, as evidenced by a slower reduction of APC/C^Cdc20^ substrates cyclin B1 and securin (Fig. 4A–B). Similar to HOIP deficiency (Fig. 1C–D) and consistent with the previously documented roles of p31^comet^ in activating APC/C^Cdc20 8^, accumulation of cyclin B1 and securin was found in p31^comet^-depleted cells (Fig. 4C–D). Moreover, analogous to HOIP knockout, deletion of p31^comet^ in HCT116 cells led to extended mitotic exit and slower degradation of APC/C^Cdc20^ substrates cyclin B1 and securin (Fig. 4E). Next, we compared the mitotic duration of control (sgGFP) and sgHOIP H2B-YFP expressing HeLa cells using time-lapse microscopy. Compared with the mean mitotic duration of 67.9 min of sgGFP cells, *HOIP* KO cells exhibited significantly prolonged mitosis with a mean duration of 101.1 min (Fig. 4F–G). Notably, a close examination of sgHOIP-HeLa cells unveiled an increase of chromosome lagging in cells undergoing cytokinesis, further suggesting elevated chromosomal instability in HOIP-deficient cells (Figures S4A). Chromosome missegregation activates the DNA damage response [38]. Indeed, p-S139-H2 A.X (γH2 A.X) was increased in sgHOIP-HCT116 cells (Figures S4B). Moreover, in line with this observation, HOIP depletion in HeLa cells resulted in increased number of cells in G2/M as evidenced by FACS analysis (Figures S4 C-E). Consistent with the difference in mitotic durations between control and sgp31 HeLa/H2B-YFP cells (Fig. 4H–I). Intriguingly, there were substantially more cells that had mitotic durations longer than 120 min in both sgHOIP and sgp31 cells, suggesting that both proteins accelerate mitotic progression.

### Ubiquitination-deficient p31^comet^ mutant is less potent in promoting mitotic progression

**Fig. 5:**
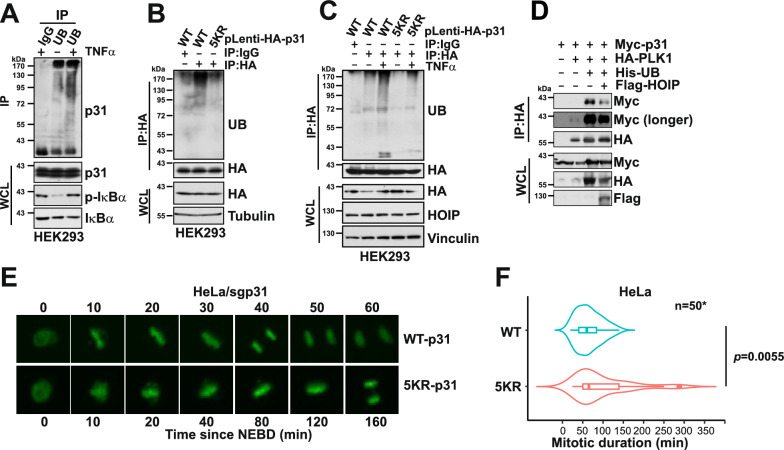
Cells expressing a linear ubiquitination-deficient p31^comet^ exhibit prolonged mitosis. **A.** Immunoblot (IB) analysis of whole cell lysates (WCL) and anti-ubiquitin immunoprecipitates (IP) derived from HEK293 cells. Cells were treated with 50 ng∙ml^−1^ TNFα for 10 min as indicated before harvest. **B**. IB analysis of WCL and anti-HA IP derived from HEK293 cells. **C**. IB analysis of WCL and anti-HA IP derived from HEK293 cells. Cells were treated with 50 ng∙ml^−1^ TNFα for 10 min as indicated before harvest. **D**. IB analysis of WCL and anti-HA IP derived from 293 T cells transfected with Myc-p31^comet^, HA-PLK1, His-UB and Flag-HOIP as indicated. **E–F**. Time-lapse microscopy of sgp31-H2B-YFP-HeLa cells stably expressing WT or 5KR-p31^comet^. **E** Representative images showing that 5KR-p31-HeLa cells took a significantly longer time to complete the mitosis. **F** Quantification of the recorded mitotic duration times (minutes) of WT- and 5KR-p31^comet^ expressing cells, n = 50, Student’s t-test. (See also Figure S5)

The results above support the notion of positive regulation of mitotic progression by HOIP, likely through HOIP-mediated linear ubiquitination of p31^comet^ (Fig. 3). We thus sought to examine if TNFα, which activates HOIP, is capable of promoting p31^comet^ poly-ubiquitination. In HEK293 cells, acute activation of the NF-κB pathway by TNFα, stimulated p31^comet^ endogenous poly-ubiquitination (Fig. 5A). In addition, the mutant 5KR-p31, which is p31-HeLa cells took a significantly (Fig. 3F), showed decreased poly-ubiquitination compared with the WT (Fig. 5B). Moreover, while TNFα provoked the ubiquitination of WT-p31 in HEK293 cells, it failed to do so in cells expressing 5KR-p31 (Fig. 5C). p31^comet^ exerts its scaffolding role in mitosis mainly through its displacement of open MAD2 from the MCC complex, thereby restraining the activity of the mitotic checkpoint [14].

Linear polyubiquitination often functions to facilitate or prevent the interaction of substrate proteins with their binding partners. Since the lysine residues ubiquitinated by HOIP are mostly located on the p31^comet^ disordered C-terminal region, rather than the MAD2-p31^comet^ interface (Fig. 3E), we reasoned that the poly-ubiquitin chains assembled on p31^comet^ may not directly interfere with MAD2-p31^comet^ binding. Indeed, co-expressing His-Ub and Flag-HOIP did not interfere with MAD2-p31^comet^ binding (Figure S5 A). PLK1 was reported as an upstream kinase that phosphorylated p31^comet^ and prevented its binding to MCC [39]. PLK1 phosphorylation site identified on p31^comet^ is S102 [39], which resides right at the flexible region where p31^comet^ is ubiquitinated by HOIP (Fig. 3E). Therefore, we hypothesize that polyubiquitination of p31^comet^ prevents its binding to PLK1 and hence maintains p31^comet^’s activity in compromising the MCC (Figs. 5D and S5B). In support of this, sgp31-HeLa cells expressing the ubiquitination-deficient 5KR-p31 mutant failed to rescue the aberrantly extended mitotic duration compared with the WT (Fig. 5E–F). Furthermore, similar to HOIP depletion, known APC/C^Cdc20^ substrates securin and cyclin B1 were upregulated in 5KR-p31^comet^-expresisng HCT116 cells (Figure S5 C), indicating a less active APC/C^Cdc20^ in cells harboring ubiquitination-deficient p31^comet^. In summary, our data presented here reveal a molecular mechanism through which the proinflammatory cytokine-directed signaling modules crosstalk with the mitotic checkpoint system. Such crosstalk could contribute to the observed genomic instability caused by inflammation in tumor cells [40].

## Discussion and conclusions

In cells, DNA damage can be generated by endogenous and exogenous factors [41]. Exogenous factors include UV/ionizing radiation (IR) and DNA damaging small molecules [41]. Besides a direct DNA-breaking effect, IR also indirectly introduces DNA damage through the generation of free radicals [42]. Free radicals can also be produced endogenously as metabolism byproducts [42]. Another major factor that contributes to DNA damage from within the cells is replication and chromosome segregation errors, which occur during the S and M phases, respectively [41]. In contrast to deletion and insertion from DNA replication mistakes, chromosome missegregation often results in double-strand DNA break (DSB) [41].

In the tumor microenvironment (TME), proinflammatory cytokines induce DNA damage likely through the accumulation of free radicals, mainly in the form of reactive oxygen and nitrogen species (RONS) [42]. The production of RONS is an important mechanism used by immune cells to target pathogens. On the other hand, prolonged exposure of somatic cells to RONS from chronic inflammation damages normal tissue and is considered a major risk factor leading to carcinogenesis [43]. Proinflammatory cytokines stimulate iNOS and COX2 expression which enhance the production of RONS [44]. RONS, in turn, promotes the transcription of iNOS and COX2 [44]. This positive feedback loop is vital for mounting a rapid and strong inflammatory response to infection, whereas it could also cause DNA damage to the host cells [45]. With an intact DNA damage repair pathway, normal cells are usually capable of coping with genomic stress imposed by infection and inflammation. Once the barrier of guarding DNA damage repair is breached such as through *TP53* loss, the inflammatory response could be oncogenic [43]. Beyond the interplay between proinflammatory cytokines and RONS, there is mounting research interest concerning the crosstalk between proinflammatory cytokines and genomic instability in tumor cells [46].

Despite several mechanisms to maintain genomic stability during cell cycle progression, DNA lesions are inevitably introduced in the S and M phases [47]. In normal cells, most of these damages could be successfully repaired via the engagement of DNA damage repair pathways including mismatch repair (MMR), nucleotide excision repair (NER), homologous recombination (HR), and non-homologous end joining (NHEJ) pathways [48]. In the scenario where the repair is unsuccessful, innate immune response and apoptotic pathways will be initiated to eliminate the DNA-damaged cells [48]. In tumor cells, due to defects in both DNA repair and apoptotic pathways, DNA lesions accumulate, which subsequently contribute to genomic instability and aneuploidy [48]. In sporadic tumors, oncogene-induced replication stress is an early driver of genomic instability [49]. There is increasing effort to investigate how the factors from the TME, such as proinflammatory cytokines, drive genomic instability during tumor evolution [50]. Although the aforementioned cytokine-induced RONS production could be responsible for small DNA lesions such as mutations and insertions, it remains elusive whether the inflammatory signals could also be a cause for chromosomal instability (CIN) such as chromosome gain/loss and micronuclei [28].

A compromised spindle assembly checkpoint (SAC) is the major cause of CIN [51]. Genetic evidence from cells and model animals suggests that defects in mitotic checkpoint genes often result in CIN [51]. For example, MAD2 depletion in cells led to chromosome missegregation and eventually cell death [52]. In mice, germline Mad2 knockout is embryonic lethal. Embryonic cells without Mad2 experienced widespread chromosome missegregation and apoptosis [53]. Intriguingly, overexpression of Mad2 in mice also results in CIN and neoplasia [54]. These findings indicate that a balanced mitotic checkpoint function is vital for maintaining genomic stability and cell fitness, even for tumor cells. Hence, it is not surprising that MAD2, BUBR1, and/or BUB3 are not frequently amplified, deleted, or mutated in human cancers (cBioPortal.org). In this regard, delicate and tunable mechanisms are likely responsible for the compromised mitotic checkpoint in tumor cells without a drastic perturbation of the mitotic machinery.

In this study, we demonstrate that p31^comet^, which restrains MAD2 and MCC function during M phase [11, 12], is subjected to linear poly-ubiquitination by HOIP. HOIP plays a pivotal role in NF-κB pathway activation [19], and is provoked in response to pathogen or cytokine stimuli as part of the cellular innate immune system [55]. Our findings that HOIP ubiquitinates p31^comet^ after proinflammatory cytokine treatment unveil a previously unknown link between cytokine response and mitotic checkpoint regulation. Different from canonical LUBAC substrates such as NEMO [37], the ubiquitination of p31^comet^ appears to require only HOIP, but not HOIL-1L or SHARPIN. This notion is supported by the observation that knockdown of neither HOIL-1L nor SHARPIN altered the levels of APC/C^Cdc20^ substrates (Figs. 1C–H and S1G-N). HOIL-1L and SHARPIN play crucial roles in activating HOIP E3 ligase activity during NF-κB activation [56]. Therefore, it will be interesting to further explore the molecular mechanisms by which HOIP functions independently of the LUBAC complex to catalyze the linear ubiquitination of p31^comet^. In search of the potential functions of p31^comet^ linear ubiquitination, we observed a decreased binding between p31^comet^ and PLK1 (Figs. 5D and S5B), but not with MAD2 (Figure S5A), when p31^comet^ is ubiquitinated by HOIP. We acknowledge that there may be other, yet undiscovered, functions of p31^comet^ linear ubiquitination. Future investigations are warranted to further characterize this newly discovered regulation of p31^comet^. Different from the K48-linkage, linear polyubiquitin chains function as scaffolds for the recruitment and activation of NF-κB pathway kinases such as the IKK kinase complex [19]. Following this direction, it is tempting to postulate that the M1-linked polyubiquitin chains assembled on p31^comet^ may help to recruit and activate NF-κB pathway kinases, which may contribute to the observed activation of NF-κB during mitotic arrest [57].

Exposed DNA due to chromosome missegregation activates the cGAS-STING pathway [58]. Recent studies highlight the important roles of this innate immune surveillance mechanism in detecting naked DNA from chromatin separation errors during mitosis [59–62]. However, it remains largely unclear whether and how the innate immune response regulates mitotic progression. A recent report showed that LUBAC promoted the linear ubiquitination of the kinetochore protein CENP-E, which facilitated its kinetochore tethering [31]. Our results here elucidate an extra layer of regulation of the mitotic checkpoint during metaphase to anaphase transition.

## Supplementary Information

Additional file 1: Figure S1. APC/C^Cdc20^ substrates are accumulated in HOIP-depleted cells. A-C. Immunoblot (IB) analysis of whole-cell lysates (WCL) derived from HEK293 (A) and HeLa (B)  cells. Cells were arrested in mitosis using 300 nM nocodazole for 16 h. Mitotic cells were collected by mechanical shake-off and released back into the cell cycle in media without nocodazole. Cells were harvested at the indicated time points after nocodazole release. When indicated, cells were treated with 50 ng∙ml-1 TNFα when released from nocodazole release. (C) The relative intensities of securin bands were calculated by normalizing to the Vinculin loading control and then to the time 0. D-E. IB analysis of WCL derived from HeLa cells (D). Cells were arrested in mitosis using 300 nM nocodazole for 16 h. Mitotic cells were collected by mechanical shake-off and released back into the cell cycle in media without nocodazole. Cells were harvested at the indicated time points after nocodazole release. When indicated, cells were treated with 40 ng∙ml-1 IL-1β when released from nocodazole release. (E) The relative intensities of securin bands were calculated by normalizing to the Vinculin loading control and then to the time 0. F. IB analysis of WCL derived from HeLa cells. Cells were arrested in mitosis using 300 nM nocodazole for 16 h. Mitotic cells were collected by mechanical shake-off and released back into the cell cycle in media without nocodazole. Cells were harvested at the indicated time points after nocodazole release. When indicated, cells were treated with 5 ng∙ml-1 or 50 ng∙ml-1 TNFα when released from nocodazole release.  G-I. IB analysis of WCL derived from HEK293 (G), DLD1 (H), and U2-OS (I) cells transduced with shScr (as the negative control) or the indicated lentiviral shHOIP constructs. The transduced cells were selected with 1 g∙ml-1 puromycin for 72 h before harvest. J. IB analysis of WCL derived from HCT116 cells transduced with sgGFP (as the negative control) or sgHOIP lentiviral constructs. The cells were treated with 50 ng∙ml-1 TNFα for 16 h before harvest. K-L. IB analysis of WCL derived from DLD1 (K) and HEK293 (L) cells transduced with shScr (as the negative control) or the indicated lentiviral shHOIL-1L constructs. The transduced cells were selected with 1 g∙ml-1 puromycin for 72 h before harvest. M-N. IB analysis of WCL derived from DLD1 (M) and HEK293 (N) cells transduced with shScr (as the negative control) or the indicated lentiviral shSHARPIN constructs. The transduced cells were selected with 1 g∙ml-1 puromycin for 72 h before harvest.

Additional file 2: Figure S2. HOIP specifically binds to p31^comet^. A-B. Immunofluorescent staining of HeLa cells using anti-HOIP and anti-p31comet antibodies, DAPI was used for DNA staining. Representative images shown here are from mitotic HeLa cells (A) and interphase HeLa cells (B).

Additional file 3: Figure S3. HOIP promotes linear ubiquitination of NEMO. A. Bacterially purified recombinant His-NEMO proteins were incubated with E1, E2, ubiquitin (UB), immune-purified WT and catalytic inactive (R2-CS) Flag-HOIP as indicated at 30 °C for 60 min before resolved by SDS-PAGE and IB analysis. 

Additional file 4: Figure S4. Delayed mitotic exit is observed in HOIP-depleted cells. A. Time-lapse microscopy of sgGFP and sgHOIP HeLa cells stably expressing a H2B-YFP retroviral construct. Representative images with higher resolution from the time point 60 min for sgGFP cells and the 120 min for sgHOIP cells shown in Figure 4F. B. IB analysis of WCL derived from HCT116 cells transduced with sgGFP (as the negative control) or sgHOIP lentiviral constructs. C-E. FACS analysis of sgGFP-HeLa (C) and sgHOIP-HeLa (D), which were fixed and stained with propidium iodide before being sorted using a BD FACSCanto II flow cytometer. Cell cycle phases were analyzed using FlowJo and compared in (E).

Additional file 5: Figure S5. Cells expressing a linear ubiquitination-deficient p31^comet^ exhibit prolonged mitosis. A. IB analysis of WCL and anti-HA IP derived from 293T cells transfected with Myc-p31comet, HA-MAD2, His-UB and Flag-HOIP as indicated. B. IB analysis of WCL and anti-HA IP derived from nocodazole arrested HeLa/HA-p31 cells. Cells were arrested in mitosis using 300 nM nocodazole for 16 h. Mitotic cells were collected by mechanical shake-off. When indicated, cells were treated with 50 ng∙ml-1 TNFα for 10 min before harvest. C. sgp31-H2B-YFP-HCT116 cells stably expressing WT or 5KR- p31^comet^ were treated with 50 ng∙ml-1 TNFα for 16 h before harvest for WB.

## Data Availability

The datasets used and/or analysed during the current study are available from the corresponding author on reasonable request.
