# Supplementary figures and images for: Linear ubiquitination of p31comet by HOIP couples cytokine response with mitotic regulation
Source: Cell Biosci. 2025 Jun 3;15:75. doi: 10.1186/s13578-025-01416-8 (PMC12135451; doi:10.1186/s13578-025-01416-8)

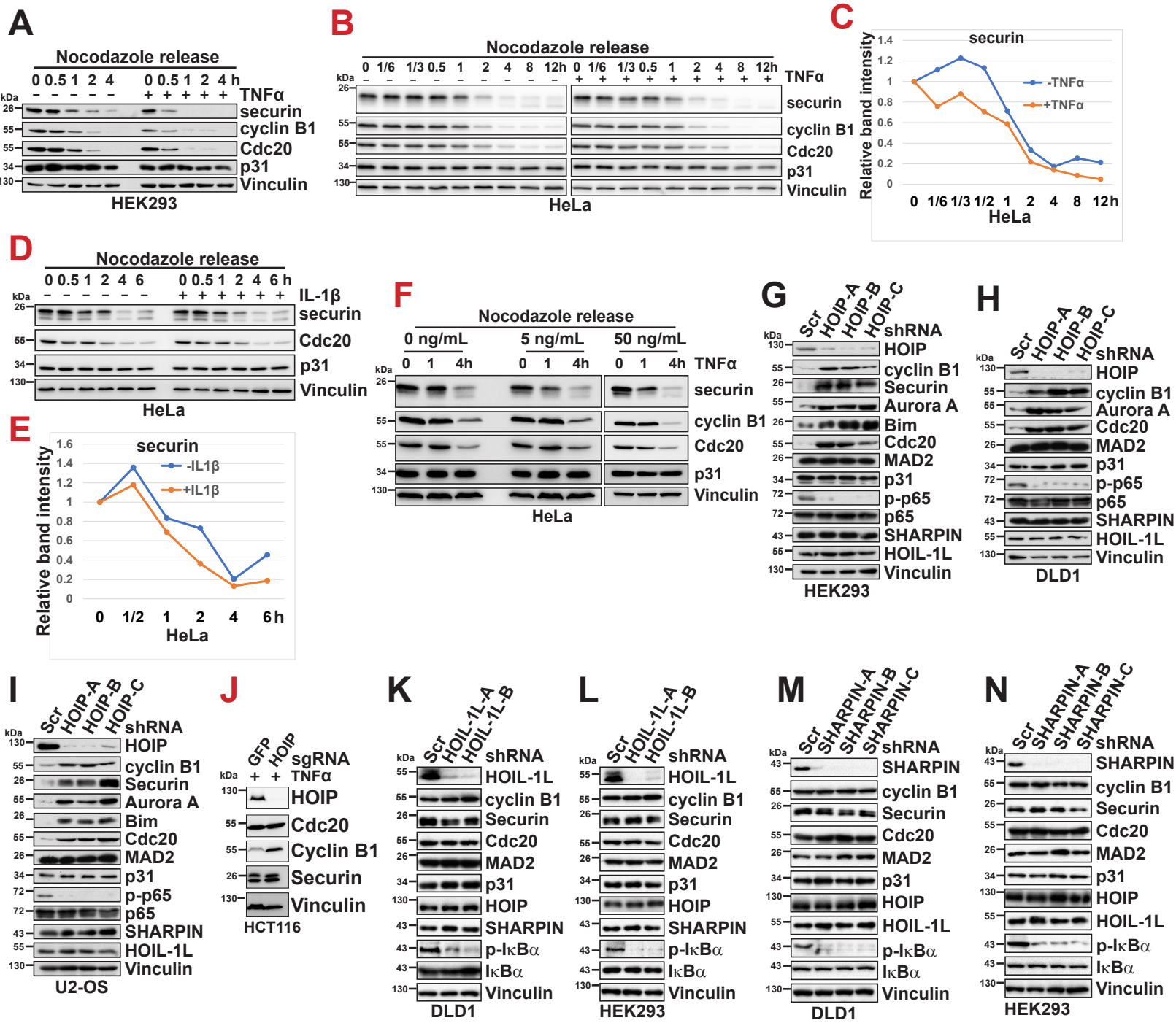

Figure S1

Supplement: Supplementary file 1 — Additional file 1: Figure S1. APC/CCdc20 substrates are accumulated in HOIP-depleted cells. A-C. Immunoblot (IB) analysis of whole-cell lysates (WCL) derived from HEK293 (A) and HeLa (B) cells. Cells were arrested in mitosis using 300 nM nocodazole for 16 h. Mitotic cells were collected by mechanical shake-off and released back into the cell cycle in media without nocodazole. Cells were harvested at the indicated time points after nocodazole release. When indicated, cells were treated with 50 ng∙ml-1 TNFα when released from nocodazole release. (C) The relative intensities of securin bands were calculated by normalizing to the Vinculin loading control and then to the time 0. D-E. IB analysis of WCL derived from HeLa cells (D). Cells were arrested in mitosis using 300 nM nocodazole for 16 h. Mitotic cells were collected by mechanical shake-off and released back into the cell cycle in media without nocodazole. Cells were harvested at the indicated time points after nocodazole release. When indicated, cells were treated with 40 ng∙ml-1 IL-1β when released from nocodazole release. (E) The relative intensities of securin bands were calculated by normalizing to the Vinculin loading control and then to the time 0. F. IB analysis of WCL derived from HeLa cells. Cells were arrested in mitosis using 300 nM nocodazole for 16 h. Mitotic cells were collected by mechanical shake-off and released back into the cell cycle in media without nocodazole. Cells were harvested at the indicated time points after nocodazole release. When indicated, cells were treated with 5 ng∙ml-1 or 50 ng∙ml-1 TNFα when released from nocodazole release. G-I. IB analysis of WCL derived from HEK293 (G), DLD1 (H), and U2-OS (I) cells transduced with shScr (as the negative control) or the indicated lentiviral shHOIP constructs. The transduced cells were selected with 1 g∙ml-1 puromycin for 72 h before harvest. J. IB analysis of WCL derived from HCT116 cells transduced with sgGFP (as the negative control) or s [file 13578_2025_1416_MOESM1_ESM.pdf]

**A**

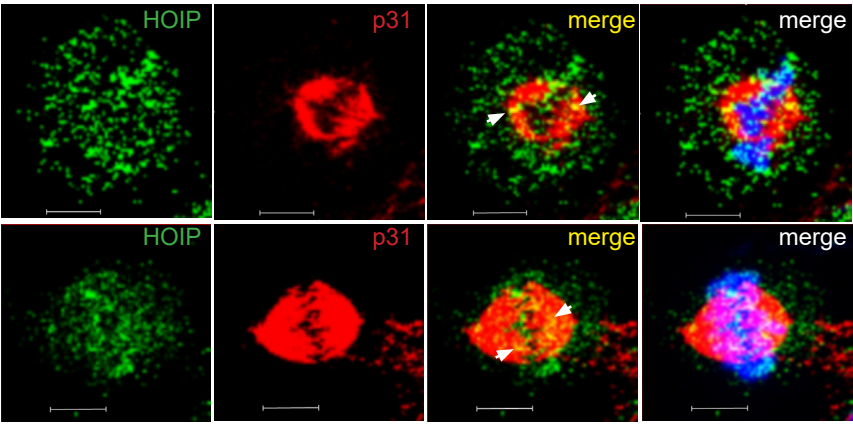

HeLa

**B**

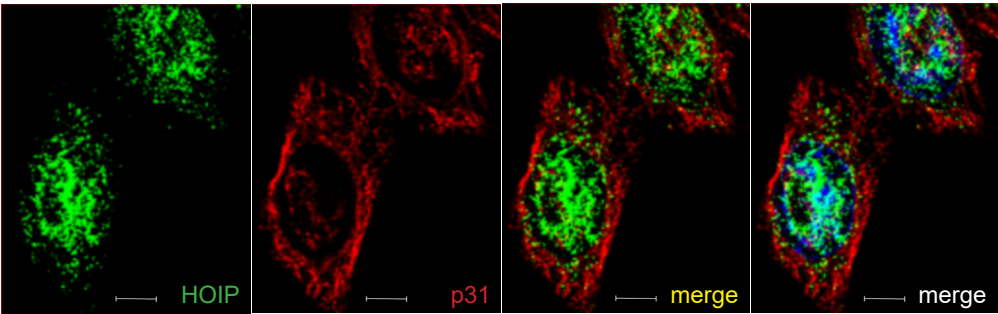

HeLa

**Figure S2**

Supplement: Supplementary file 2 — Additional file 2: Figure S2. HOIP specifically binds to p31comet. A-B. Immunofluorescent staining of HeLa cells using anti-HOIP and anti-p31comet antibodies, DAPI was used for DNA staining. Representative images shown here are from mitotic HeLa cells (A) and interphase HeLa cells (B). [file 13578_2025_1416_MOESM2_ESM.pdf]

**A**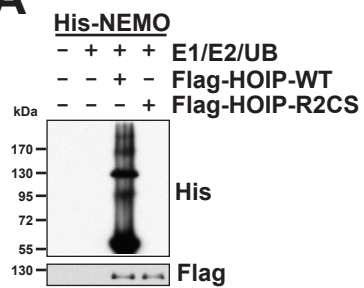**Figure S3**

Supplement: Supplementary file 3 — Additional file 3: Figure S3. HOIP promotes linear ubiquitination of NEMO. A. Bacterially purified recombinant His-NEMO proteins were incubated with E1, E2, ubiquitin (UB), immune-purified WT and catalytic inactive (R2-CS) Flag-HOIP as indicated at 30 °C for 60 min before resolved by SDS-PAGE and IB analysis. [file 13578_2025_1416_MOESM3_ESM.pdf]

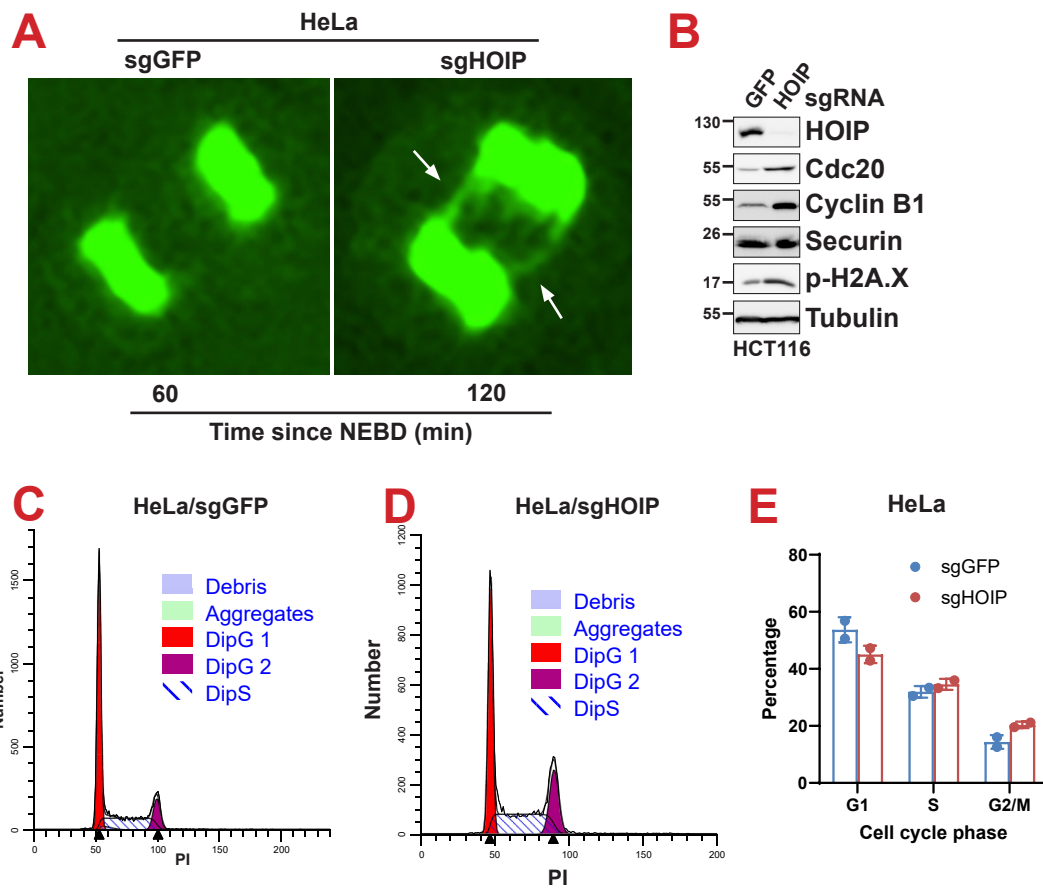

Figure S4

Supplement: Supplementary file 4 — Additional file 4: Figure S4. Delayed mitotic exit is observed in HOIP-depleted cells. A. Time-lapse microscopy of sgGFP and sgHOIP HeLa cells stably expressing a H2B-YFP retroviral construct. Representative images with higher resolution from the time point 60 min for sgGFP cells and the 120 min for sgHOIP cells shown in Figure 4F. B. IB analysis of WCL derived from HCT116 cells transduced with sgGFP (as the negative control) or sgHOIP lentiviral constructs. C-E. FACS analysis of sgGFP-HeLa (C) and sgHOIP-HeLa (D), which were fixed and stained with propidium iodide before being sorted using a BD FACSCanto II flow cytometer. Cell cycle phases were analyzed using FlowJo and compared in (E). [file 13578_2025_1416_MOESM4_ESM.pdf]

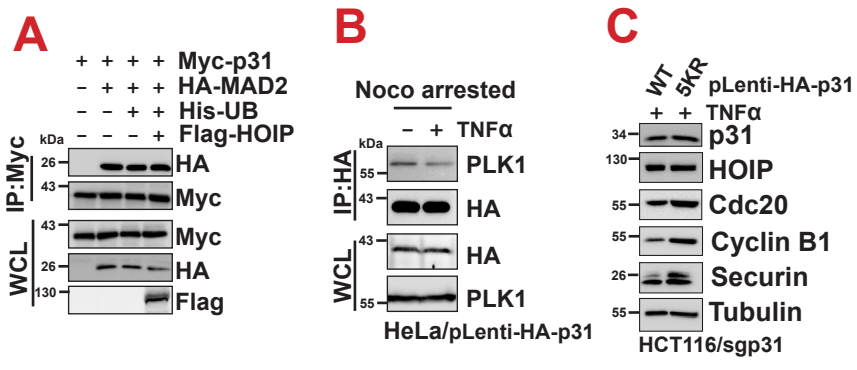

Figure S5

Supplement: Supplementary file 5 — Additional file 5: Figure S5. Cells expressing a linear ubiquitination-deficient p31comet exhibit prolonged mitosis. A. IB analysis of WCL and anti-HA IP derived from 293T cells transfected with Myc-p31comet, HA-MAD2, His-UB and Flag-HOIP as indicated. B. IB analysis of WCL and anti-HA IP derived from nocodazole arrested HeLa/HA-p31 cells. Cells were arrested in mitosis using 300 nM nocodazole for 16 h. Mitotic cells were collected by mechanical shake-off. When indicated, cells were treated with 50 ng∙ml-1 TNFα for 10 min before harvest. C. sgp31-H2B-YFP-HCT116 cells stably expressing WT or 5KR- p31comet were treated with 50 ng∙ml-1 TNFα for 16 h before harvest for WB. [file 13578_2025_1416_MOESM5_ESM.pdf]
